# Reliability and validity testing of the Chinese version of the self-directed learning scale among middle school students

**DOI:** 10.3389/fpsyg.2025.1642596

**Published:** 2025-11-25

**Authors:** Fei Cheng, Mei Foong Ang, Yana An, Chuan Li, Jian Yu

**Affiliations:** 1 School of Education, Hebei Normal University, Shijiazhuang, China; 2 Department of Music, Faculty of Human Ecology, Universiti Putra Malaysia, Selangor, Malaysia; 3 Shijiazhuang Finance and Trade School, Shijiazhuang, China; 4 School of Teacher Education, Hebei Normal University, Shijiazhuang, China; 5 Normal Practice Guidance Center, Hebei Normal University, Shijiazhuang, China

**Keywords:** self-directed learning, scale translation and adaptation, construct validity, exploratory and confirmatory factor analysis, cross-cultural validation, Chinese secondary education, adolescent learner autonomy

## Abstract

**Introduction:**

This study examined the psychometric properties of the Chinese version of the Self-Directed Learning Scale (SDLS) among 979 middle school students in China.

**Methods:**

The scale was translated and culturally adapted using Brislin’s and Beaton’s cross-cultural procedures. Analyses conducted with SPSS 29.0 and Amos 26.0 included assessments of internal consistency, content validity, and structural validity.

**Results:**

The SDLS demonstrated strong reliability (Cronbach’s α = 0.882; McDonald’s ω = 0.887; CR = 0.887) and excellent content validity (S-CVI/Ave = 0.980). Exploratory factor analysis supported a one-factor structure (KMO = 0.895), which was confirmed by confirmatory factor analysis (χ^2^/df = 4.422, CFI = 0.951, TLI = 0.927, RMSEA = 0.084).

**Discussion:**

The findings indicate that the Chinese SDLS is a reliable and valid instrument for assessing self-directed learning in adolescents. It provides a practical tool for learner profiling and educational planning within China’s performance-oriented yet increasingly student-centered context.

## 1 Introduction

Self-directed learning has received considerable attention in recent years (e.g., Beach, 2017; Hiemstra, 2013; Hwang and Oh, 2021; Zhang and Yang, 2023). It is believed to encompass skills such as setting learning goals, identifying educational resources, and evaluating learning outcomes (Zhoc and Chen, 2016). Knowles (1975) defined self-directed learning as “the process by which an individual, with or without the help of others, actively diagnoses learning needs, sets learning goals, determines human and material resources for learning, selects and implements appropriate learning strategies, and evaluates learning outcomes.” This was once most commonly used definition in many studies (O’Shea, 2003).

However, this definition has been challenged for emphasizing the skills and abilities required for individuals to participate in learning experiences (Zhoc and Chen, 2016). Possessing knowledge and skills alone may not ensure a person’s sustained engagement in learning throughout their life (Little, 2000; Macaskill and Denovan, 2013; Oddi, 1987). Persistence is a psychological factor that is not necessarily influenced by skills alone (Macaskill and Denovan, 2013).

Guglielmino’s (1977) Self-Directed Learning Readiness Scale (SDLRS) and Oddi (1986) Continuous Learning Inventory (OCLI) are two major instruments developed from the personality perspective of self-directed learning (Harvey et al., 2006; Svedberg, 2010). The SDLRS comprises 58 items that measure an individual’s readiness for self-directed learning. The scale includes eight factors: (1) openness to learning opportunities; (2) self-concept as an effective learner; (3) initiative and independence in learning; (4) informed acceptance of responsibility for one’s own learning; (5) love of learning; (6) creativity; (7) future orientation; and (8) the ability to use basic study and problem-solving skills. By contrast, the OCLI comprises 24 items that measure three characteristics of self-directed continuous learners: (1) proactive versus reactive drive (initiating and persisting in learning without external reinforcement); (2) cognitive openness versus defensiveness (adaptability, flexibility, and receptivity to change, and willingness to take risks); and (3) commitment to learning versus apathy or aversion to learning (actively pursuing and enjoying learning for its own sake) (Zhoc and Chen, 2016).

Despite the popularity of these instruments, they have faced several criticisms. For instance, Field (1989) and Bonham (1991) criticized the SDLRS for its weak conceptual foundation, arguing that it measures a homogeneous construct of love and enthusiasm for learning rather than actual readiness. As for the OCLI, Landers (1990) criticized it for weak internal reliability.

Adopting the personality trait perspective, Brockett (1983) described self-directed learning as a tendency to engage in learning activities in which individuals assume personal responsibility for designing and executing learning processes independently, without external guidance from teachers, parents, or peers. He suggested that self-directed learning reflects a person’s preference for managing their learning process, including planning, implementation, evaluation, goal orientation, and the ability to work independently or collaboratively with minimal guidance.

Lounsbury and Gibson (2006) developed the Self-Directed Learning Scale (SDLS) based on Brockett’s (1983) theory. The SDLS is part of a personality trait measurement system for adolescents and adults. It is a unidimensional scale comprising only 10 items and is particularly valued for its simplicity. Additionally, it demonstrates strong psychometric properties and has been applied to university, junior high school, and senior high school samples. Furthermore, the scale has been found to correlate with several related personality traits, such as the Big Five Personality Traits of Openness to experience, conscientiousness, neuroticism, optimism, and life and college satisfaction (Zhoc and Chen, 2016).

The SDLS demonstrated strong psychometric properties across various educational and cultural contexts (e.g., Demircioğlu et al., 2018; Lounsbury et al., 2009; Zhoc and Chen, 2016). Zhang and Yang (2023) also confirmed its robust measurement properties in a sample of Chinese college students. However, no studies have evaluated the SDLS among middle school students in the Chinese context. This gap is noteworthy because adolescence is a critical developmental stage when learners transition from external guidance to more autonomous modes of learning (Eccles and Roeser, 2011). Understanding SDL at this stage is particularly important in China, where middle school students experience high-stakes examinations and intensive academic pressure that shape their motivation and learning behaviors (OECD, 2019).

Furthermore, the Chinese cultural and educational context provides unique conditions for examining SDL. Rooted in Confucian heritage culture, Chinese education emphasizes effort, discipline, and respect for authority, often leading to teacher-centered classrooms (Li, 2012). This orientation may limit students’ ability to exercise independent decision-making in learning. Similarly, China’s collectivist cultural values contrast with the individualistic assumptions underlying many Western SDL theories (Hofstede, 2001). Such cultural distinctions raise important questions about the cross-cultural validity of SDL instruments and whether Western conceptualizations of SDL can be meaningfully applied to Chinese adolescents.

Previous studies have consistently reported the SDLS to be reliable and valid; however, its factor structure remains unclear. While most studies using confirmatory factor analysis have supported a single-factor model (e.g., Lounsbury et al., 2009; Zhoc and Chen, 2016), Zhang and Yang (2023) identified a bifactor structure in a sample of Chinese college students.

Therefore, although prior research has proved the reliability and validity of the SDLS, there are still important research gaps. Specifically, applicability of SDLS to Chinese middle school students has not been tested, despite the distinctive cultural and educational characteristics of this population. Moreover, the underlying factor structure of the SDLS continues to be debated. To address these gaps, the present study aims to (1) evaluate the psychometric properties of the SDLS among Chinese middle school students and (2) examine its factor structure in this population.

## 2 Materials and methods

### 2.1 Participants

The participants were 1,057 students from six middle schools in Hebei Province, China. The data collection protocol was approved by the Human Research Ethics Committee of the first author’s university, ensuring that participants had the right to access information about the study, remain anonymous, and withdraw at any time. Before conducting the study, we obtained informed consent from all relevant parties, including schools, teachers, and parents.

At the outset, the researchers explained the study purpose to the participants and clarified their rights, including data confidentiality and the right to withdraw at any stage. The participants were encouraged to respond honestly to the questionnaire and were given the opportunity to ask questions.

The data cleaning process included three steps: Adding two polygraph items to the questionnaire, requiring a minimum average response time of 2 s per item, and ensuring that repeated answers did not exceed half of the total number of items (Curran, 2016; Soland et al., 2019). A total of 979 valid questionnaires were collected, with an effective response rate of 92.6%.

More than half of the sample were boys (57.1%, *n* = 559) [girls (42.9%, *n* = 420)]. Regarding residential background, 53.2% of participants were from rural areas (*n* = 521) and 46.8% from urban areas (*n* = 458). In terms of grade level, 22.4% were in the first year of junior high school (*n* = 219), 24.6% in the second year (*n* = 241), and 16.9% in the third year (*n* = 165). At the high school level, 12.5% were in the first year (*n* = 122), 12.6% in the second year (*n* = 124), and 11.0% in the third year (*n* = 108).

### 2.2 Instrument: Chinese version of self-directed learning scale (SDLS)

For a measurement instrument to be used cross-culturally, it must be translated accurately and culturally adapted to preserve its conceptual validity across different contexts (Beaton et al., 2000). The English version of the SDLS was translated into Chinese following Brislin (1970) translation model and Beaton et al. (2000) guidelines for cross-cultural adaptation. The process included five steps: (1) Forward Translation: Two bilingual translators whose native language is Chinese independently translated the SDLS from English into Chinese. Both had graduate-level training in language studies but were unfamiliar with the scale. The two independent translations produced T1 and T2. (2) Synthesis: The translators compared T1 and T2 and reconciled discrepancies to produce a synthesized version (ST1&2). Differences in wording were resolved through discussion, prioritizing conceptual equivalence over literal translation. (3) Back Translation: Two bilingual researchers (one psychologist, one language expert), blinded to the original SDLS, independently translated ST1&2 back into English, generating BT1 and BT2. This step ensured that the Chinese version retained the meaning of the original scale. (4) Expert Committee Review: A panel of three bilingual psychologists and three bilingual middle school educators reviewed all translations (T1, T2, ST1&2, BT1, BT2). The panel evaluated semantic, idiomatic, experiential, and conceptual equivalence, resolved discrepancies, and finalized a pre-final Chinese version of the SDLS. (5) Pretesting (Cognitive Debriefing): Thirty middle school students (aged 14–16; from the second year of junior high school to the first year of high school; 15 males, 15 females) completed the pre-final SDLS. Item clarity and comprehensibility were assessed through a combination of cognitive interviews and questionnaires. During pretesting, we focused on items containing abstract terms such as “initiative” and “goal-setting.” Cognitive interviews confirmed that most students could understand these concepts when contextualized with simple examples. On the basis of the feedback, the final Chinese version of the SDLS was confirmed.

Certain items required careful consideration to ensure both conceptual equivalence and cultural appropriateness in the Chinese context. Below we summarize the main issues encountered and how they were addressed.

Career Orientation (Item 5): The original item (“I view self-directed learning based on my own initiative as very important for success in school and in my future career”) emphasizes long-term career development. During the pretest, several middle school students indicated that “career” (职业生涯) was a rather distant concept for their current stage. To ensure comprehensibility while preserving the original meaning, the Chinese version retained “未来职业生涯” but contextualized it within “在学校和未来职业生涯中的成功”. This formulation made the item more accessible to adolescents by first anchoring it in their immediate academic setting before extending to their future career.

Autonomy and Self-Agency (Items 6 and 7): Items related to setting personal goals (“我为要学习的内容设定自己的目标”) and taking charge of learning (“我喜欢掌控自己学习的内容和时间”) may be perceived differently in the Chinese context, where learning is often guided by teachers and curricula. Pretest interviews revealed that some students initially associated these items with teacher-assigned tasks only. To address this, the wording emphasized “自己” (my own), reinforcing the notion of personal initiative while still aligning with the collectivist educational environment.

According to the statement of the original authors, “researchers who wish to use this scale may do so without charge as long as it is not used for profit-making purposes and they cite this article.” In line with this requirement, the SDLS was translated and used in the present study solely for academic research, with appropriate citation to the original source.

The detailed administration instructions, scoring rules, and the full Chinese version of the SDLS are provided in Supplementary material.

### 2.3 Data analysis method

Data were analyzed using IBM SPSS Statistics 29.0 and Amos 26.0. Internal consistency reliability of the Chinese version of the SDLS was assessed using Cronbach’s alpha coefficient. Cronbach’s alpha is a widely used indicator of internal consistency (Bonett, 2002), with values of 0.70 and above generally indicating good reliability (George, 2011).

Content validity was evaluated using the item-level content validity index (I-CVI) and the scale-level content validity index (S-CVI). The I-CVI represents the proportion of experts who rate an item as “relevant” or “highly relevant.” Experts typically use a 4-point rating scale (e.g., 1 = not relevant; 4 = highly relevant). An I-CVI of 0.78 or higher is considered acceptable when six or more experts are involved (Lynn, 1986). The S-CVI reflects the overall content validity of the scale and can be calculated in two ways. First, the S-CVI/Ave is the average of the I-CVI values across all items, with values of 0.90 or above indicating excellent validity (Polit and Beck, 2006). Second, the S-CVI/UA represents the proportion of items that achieved universal agreement among experts (i.e., all experts rated the item as “relevant” or “highly relevant”), with a value of 0.80 or higher typically denoting strong content validity (Polit et al., 2007).

Structural validity was assessed through exploratory factor analysis (EFA) and confirmatory factor analysis (CFA). To minimize overfitting and ensure independent validation, we randomly divided the 979 valid responses into two groups: the first group (*n* = 488) for EFA and the second (*n* = 491) for CFA.

For the EFA, principal axis factoring (PAF) was used as an extraction method because it accounts for measurement error and estimates common variance. Sampling adequacy was evaluated using the Kaiser–Meyer–Olkin (KMO) test and Bartlett’s test of sphericity. A KMO value ≥ 0.60 indicates acceptable sampling adequacy, with 0.70–0.80 considered good, 0.80–0.90 great, and > 0.90 superb (Kaiser, 1974). A significant Bartlett’s test (*p* < 0.05) indicates that the correlation matrix is suitable for factor analysis (Field, 2013).

Factor retention was determined using multiple criteria, including the Kaiser criterion (eigenvalues > 1), inspection of the scree plot, and a parallel analysis with 1,000 random datasets (O’Connor, 2000). Because only one factor was extracted, rotation was not applicable. Parallel analysis compares the real data eigenvalues with those generated from random correlation matrices; only factors with real eigenvalues exceeding the 95th percentile of the random data are retained.

The CFA was then conducted using the independent subsample (*n* = 491) to validate the factor structure identified in the EFA. Model fit was evaluated using multiple indices: the chi-square to degrees of freedom ratio (χ^2^/df < 5.0) (Bentler, 1990), the root mean square error of approximation (RMSEA < 0.08), comparative fit index (CFI ≥ 0.90), normed fit index (NFI > 0.90), goodness-of-fit index (GFI ≥ 0.90), incremental fit index (IFI ≥ 0.90), and Tucker–Lewis index (TLI ≥ 0.90). Relative χ^2^, RMSEA, and three to four other indices were considered sufficient to determine the adequacy of model fit (Hair et al., 2010). The SRMR value should be < 0.08 (Hu and Bentler, 1999).

Additionally, internal consistency reliability indices beyond Cronbach’s α were computed based on the standardized factor loadings and error variances derived from the CFA. Specifically, McDonald’s ω and Composite Reliability (CR) were calculated following the procedures outlined by Raykov (1997) and Dunn et al. (2014), with values ≥ 0.70 indicating satisfactory reliability (Hair et al., 2019).

To assess convergent validity, the Average Variance Extracted (AVE) was computed using standardized factor loadings and measurement error variances according to Fornell and Larcker (1981). An AVE ≥ 0.50 is generally considered acceptable, although slightly lower values may be tolerated when reliability remains adequate (Hair et al., 2019).

Additionally, to check for common method bias (CMB), we conducted a single-factor CFA by following Podsakoff et al. (2003, 2012). The fit indices of this single-factor model were compared with those of the hypothesized measurement model to determine whether a single latent factor could account for most of the covariance among the observed items.

Furthermore, PCLOSE (test of close fit for RMSEA) and Hoelter’s critical N were reported as supplementary indices.

## 3 Results

### 3.1 Internal consistency reliability

Item-level descriptive statistics are presented in Table 1. The mean scores of the 10 items ranged from 2.890 to 3.700, with standard deviations between 0.835 and 0.917. Skewness (−0.316 to 0.031) and kurtosis (0.007–0.439) values indicated that item distributions did not substantially deviate from normality. The corrected item–total correlations (CITCs) ranged from 0.524 to 0.686, all exceeding the recommended threshold of 0.30 (Field, 2018), suggesting that each item contributed meaningfully to the overall construct.

**TABLE 1:** Descriptive statistics and corrected item–total correlations for the Chinese version of the self-directed learning scale (SDLS).

Item	Mean	SD	Skewness	Kurtosis	Corrected item–total correlation
1	3.200	0.917	–0.156	0.439	0.544
2	3.160	0.881	–0.062	0.354	0.619
3	3.260	0.899	–0.166	0.007	0.573
4	3.400	0.853	–0.253	0.439	0.665
5	3.700	0.905	–0.316	0.086	0.524
6	3.530	0.889	–0.218	0.122	0.683
7	3.520	0.876	–0.276	0.295	0.659
8	3.340	0.835	0.031	0.325	0.686
9	2.890	0.886	0.011	0.279	0.569
10	3.080	0.913	0.010	0.382	0.594

The Cronbach’s α coefficient of the Chinese version of the SDLS was 0.882, indicating good reliability and confirming the internal consistency and applicability of this scale for middle school students in the Chinese context.

### 3.2 Content validity

A panel of middle school educational psychology experts, comprising three secondary education specialists and three middle school psychology teachers, evaluated each item of the Chinese version of the SDLS. All experts were well-known educators in universities or middle schools, each with more than 15 years of experience and holding either a doctoral or a master’s degree. The item-level content validity indices (I-CVIs) ranged from 0.830 to 1.000, while the scale-level indices (S-CVI/Ave and S-CVI/UA) were 0.980 and 0.900, respectively. The panel agreed that the framework of the scale effectively measured self-directed learning and that each item accurately assessed the intended construct. These findings indicate that the scale possesses robust and satisfactory content validity, warranting further examination of its structural validity through EFA and CFA.

### 3.3 Structural validity

To minimize the risk of overfitting and to ensure independent cross-validation, we randomly divided the 979 valid responses into two groups for EFA and CFA. The randomization was performed using IBM SPSS Statistics 29.0, employing the Random Sample of Cases function, with a fixed random seed of 2023 to ensure replicability. The data were split approximately 50:50, resulting in 488 cases in the EFA subsample and 491 cases in the CFA subsample. Both subsamples maintained comparable distributions of sex, residential background, and grade level, ensuring representativeness across key demographic characteristics.

#### 3.3.1 Exploratory factor analysis

To examine the underlying factor structure of the Chinese version of the SDLS, we conducted EFA using PAF as the extraction method. Sampling adequacy was supported by a high KMO value of 0.895, and Bartlett’s test of sphericity was significant, χ^2^(45) = 1924.079, *p* < 0.001, indicating suitability for factor analysis. Table 2 presents the total variance explained. The first factor’s eigenvalue (based on the correlation matrix) was 4.725, accounting for 47.25% of the total variance.

**TABLE 2:** Total variance explained for the Chinese version of the self-directed learning scale (SDLS).

Factor	Initial eigenvalue	% of Variance	Cumulative %
1	4.725	47.245	47.245
2	1.144	11.435	58.681
3	0.820	8.199	66.880
4	0.714	7.145	74.024
5	0.578	5.783	79.808
6	0.504	5.042	84.849
7	0.438	4.378	89.228
8	0.379	3.792	93.020
9	0.370	3.703	96.723
10	0.328	3.277	100

Extraction method, principal axis factoring.

Factor retention was further evaluated using the Kaiser criterion (eigenvalues > 1), inspection of the scree plot, and a parallel analysis with 1,000 random datasets (EFA subsample: *N* = 488, 10 items). The parallel analysis results (Table 3) indicated that only the first factor’s real eigenvalue (4.725) exceeded both the random mean (1.233) and the random 95th-percentile (1.294) values, whereas all subsequent real eigenvalues were lower than their random counterparts, indicating that a single factor should be retained.

**TABLE 3:** Results of parallel analysis (1,000 iterations) for the Chinese version of the self-directed learning scale (SDLS).

Factor	Real eigenvalue	Random mean	Random 95th percentile	Retained?
1	4.725	1.233	1.294	Yes
2	1.144	1.159	1.219	No
3	0.820	1.107	1.179	No
4	0.714	1.074	1.150	No
5	0.578	1.037	1.116	No
6	0.504	1.003	1.083	No
7	0.438	0.969	1.052	No
8	0.379	0.937	1.024	No
9	0.370	0.904	0.991	No
10	0.328	0.871	0.959	No

Only the first factor exceeded both the random mean and 95th-percentile eigenvalues, supporting a one-factor solution.

**FIGURE 1:**
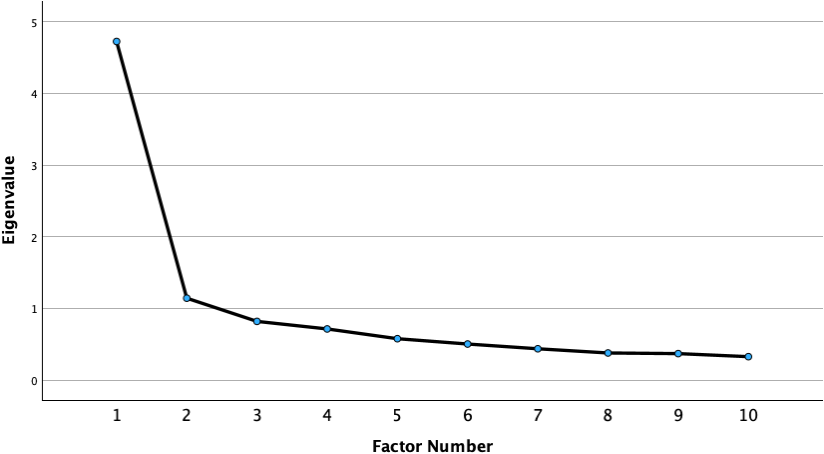
Scree plot for the exploratory factor analysis of the Chinese version of the self-directed learning scale (SDLS). The point of inflection after the first factor supports the unidimensional structure.

The scree plot (Figure 1) also shows a clear inflection after the first factor. Taken together, these results support a unidimensional factor structure for the Chinese version of the SDLS. Although the second factor’s eigenvalue (1.144) marginally exceeded 1.0, both the parallel analysis and scree plot indicated that it represented statistical noise rather than a substantively meaningful factor. Taken together, these results support a single-factor solution for the Chinese version of the SDLS.

Table 4 displays the PAF one-factor loadings for the 10 items (range: 0.514–0.760). Internal consistency for the scale in the EFA sample was good (Cronbach’s α = 0.873), and corrected item–total correlations ranged from 0.480 to 0.702 (all > 0.30), indicating that each item contributed meaningfully to the overall construct.

**TABLE 4:** Factor loadings for the Chinese version of the self-directed learning scale (SDLS).

Item	Loading (factor 1)
1	0.514
2	0.623
3	0.639
4	0.632
5	0.520
6	0.715
7	0.680
8	0.760
9	0.627
10	0.697

Extraction method, principal axis factoring; rotation, not applicable (one factor extracted).

Overall, the EFA results supported a single latent factor with high internal consistency, providing a sound empirical basis for the subsequent CFA.

#### 3.3.2 Confirmatory factor analysis

**FIGURE 2:**
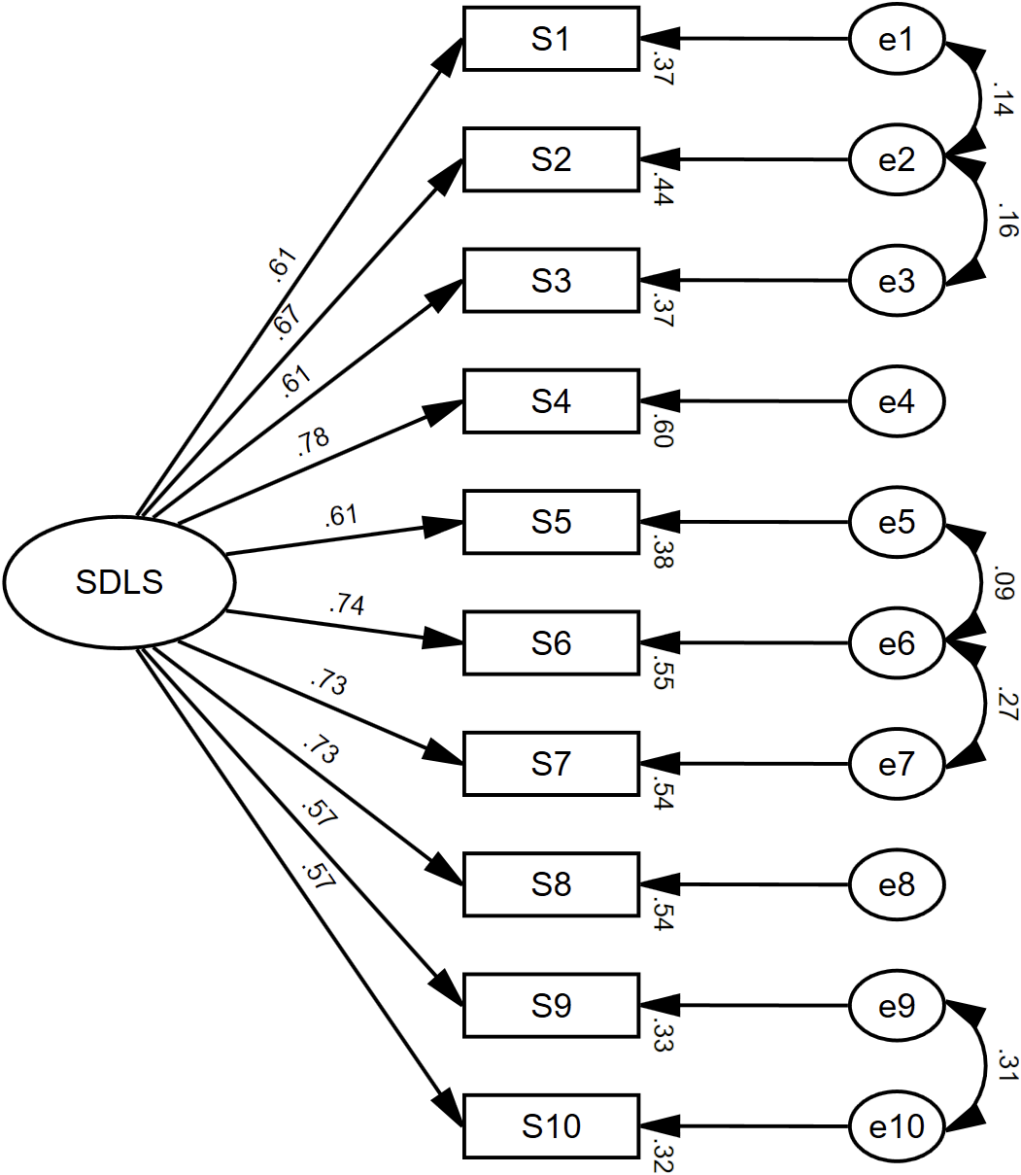
Structural equation model path diagram for the Chinese version of the self-directed learning scale (SDLS).

On the basis of the EFA results indicating a unidimensional structure, we conducted CFA on the independent subsample (*n* = 491). The hypothesized single-factor model of the SDLS was tested, with ten observed indicators (S1–S10) representing the latent construct “SDLS.” As shown in Figure 2, all standardized path coefficients from the latent factor to the observed variables ranged from 0.57 to 0.78 and were statistically significant (*p* < 0.001), indicating that each item reliably reflected the unified SDLS construct.

Several residual correlations were permitted based on theoretical and semantic justification. Items 1, 2, and 3 (e1–e2–e3) were correlated because they all describe self-initiated and self-managed learning behaviors beyond formal classroom settings. Items 5, 6, and 7 (e5–e6–e7) share similar content on goal setting and autonomy, aligning with Brockett (1983) notion of personal responsibility in self-directed learning. Items 9 and 10 (e9–e10) capture self-efficacy and intrinsic motivation for independent learning. These residual correlations have been shown to be strongly associated in previous validation studies (e.g., Zhoc and Chen, 2016; Demircioğlu et al., 2018). These correlations represent shared semantic and motivational variance rather than model misspecification, and their inclusion maintained the unidimensional nature of the SDLS.

Table 5 presents the unstandardized and standardized factor loadings, standard errors, and significance levels for the 10 items of the Chinese version of the SDLS. All items loaded significantly on the latent factor (*p* < 0.001), with standardized loadings ranging from 0.57 to 0.78, exceeding the recommended minimum of 0.50 (Hair et al., 2019). These results indicate that each item contributed meaningfully to the underlying construct of self-directed learning and support the unidimensional measurement structure.

**TABLE 5:** Standardized and unstandardized factor loadings, standard errors, and significance levels for the self-directed learning scale (SDLS).

Item	Unstandardized estimate	SE	CR	p	Standardized loading (Std. All)
1	1.000 (fixed)	–	–	–	0.61
2	1.064	0.082	12.922	<0.001	0.67
3	0.959	0.086	11.150	<0.001	0.61
4	1.208	0.092	13.187	<0.001	0.78
5	0.993	0.088	11.254	<0.001	0.61
6	1.188	0.094	12.679	<0.001	0.74
7	1.104	0.088	12.608	<0.001	0.73
8	1.083	0.086	12.668	<0.001	0.73
9	0.886	0.083	10.620	<0.001	0.57
10	0.916	0.087	10.487	<0.001	0.57

All loadings were statistically significant at *p* < 0.001. Standardized loadings (Std. All) were obtained from “Standardized Regression Weights.”

In addition, internal consistency reliability indices were calculated based on standardized CFA loadings and error variances. Both McDonald’s ω and composite reliability (CR) were satisfactory (ω = 0.887, CR = 0.887), exceeding the recommended threshold of 0.70 (Raykov, 1997; Dunn et al., 2014; Hair et al., 2019). These findings further support the internal consistency and reliability of the Chinese version of the SDLS.

Table 6 summarizes the overall model-fit indices. The revised model demonstrated an acceptable fit to the data (χ^2^/df = 4.422, CFI = 0.951, TLI = 0.927, IFI = 0.952, NFI = 0.938, RMSEA = 0.084, 90% CI [0.069, 0.098], PCLOSE = 0.000, Hoelter = 162 [0.05] and 188 [0.01], SRMR = 0.040; Hu and Bentler, 1999). According to established criteria (Bentler, 1990; Hair et al., 2010), these indices indicate a reasonably good model fit. Although the RMSEA slightly exceeded the ideal cutoff of 0.08, the CFI, TLI, and SRMR values met recommended standards, suggesting that the model was adequately specified and theoretically coherent.

**TABLE 6:** Model fit indices for the Chinese version of the self-directed learning scale (SDLS).

Fit index	Value	Recommended threshold	Reference
χ^2^	132.674	–	–
df	30	–	–
χ^2^/df	4.422	< 5.00	Bentler (1990)
CFI	0.951	≥ 0.90	Hair et al. (2010)
TLI	0.927	≥ 0.90	Hair et al. (2010)
IFI	0.952	≥ 0.90	Hair et al. (2010)
NFI	0.938	≥ 0.90	Hair et al. (2010)
SRMR	0.040	≤ 0.08	Hu and Bentler (1999)
RMSEA	0.084	≤ 0.08 (acceptable if < 0.10)	Bentler (1990)
RMSEA 90% CI	[0.069, 0.098]	–	–
PCLOSE	0.000	–	–
Hoelter (0.05)	162	>100	–
Hoelter (0.01)	188	>100	–

Furthermore, the convergent validity of the model was evaluated by calculating the AVE using the standardized factor loadings and error variances. The AVE was 0.444, which is slightly below but close to the recommended value of 0.50 (Fornell and Larcker, 1981). Given that all standardized loadings were above 0.50 and statistically significant (*p* < 0.001), the construct demonstrated adequate convergent validity (Hair et al., 2019). As the final model consisted of a single latent factor, discriminant validity could not be examined because it requires at least two constructs for inter-factor comparison (Fornell and Larcker, 1981). This is theoretically consistent with prior research suggesting that self-directed learning represents a unified construct among adolescent learners (Zhoc and Chen, 2016).

Additionally, to assess the potential influence of CMB, we conducted a single-factor CFA following the recommendations of Podsakoff et al. (2003). The hypothesized measurement model (χ^2^/df = 4.422, CFI = 0.951, TLI = 0.927, RMSEA = 0.084) was compared with a single-factor model in which all items were constrained to load onto one latent construct. The single-factor model exhibited a substantially poorer fit (χ^2^/df = 47.929, CFI = 0.000, TLI = 0.000, RMSEA = 0.309), indicating that a single factor could not account for most of the covariance among the observed variables. These results suggest that CMB was not a serious concern in the present study. This test was performed as a rough diagnostic procedure rather than a definitive control method (Podsakoff et al., 2012).

Although the present study included participants of different genders and grade levels, the sample size within each subgroup was insufficient to perform a stable multi-group CFA. Therefore, measurement invariance across subgroups (e.g., gender, grade) was not tested in this study. This limitation should be addressed in future research to further examine the stability of the SDLS across demographic groups.

Together, the results corroborated the EFA findings and confirmed the unidimensionality of the Chinese version of the SDLS, demonstrating that the adapted scale is psychometrically sound for assessing self-directed learning among middle school students in the Chinese context.

## 4 Discussion

This study provides evidence of the reliability and validity of the SDLS for application in mainland China. The results confirmed the single-factor structure of the scale. However, using a sample of mainland Chinese college students, Zhang and Yang (2023) found that the scale has a two-factor structure. This discrepancy implies that the factor structure may vary depending on sample characteristics and methodological approaches (Yu et al., 2015).

Additionally, the findings have important implications when viewed against the backdrop of Chinese educational culture. Traditional norms in China—influenced by Confucian heritage—emphasize diligence, respect for teachers, collective responsibility, and examination achievement (Yu et al., 2018). In such a context, there may be an assumption that students are less autonomous or self-initiating, and that motivation is more externally regulated (e.g., driven by parental/school expectations). Nevertheless, our results suggest that many middle school students in this sample demonstrate self-directed learning attitudes and behaviors, even within a structured and academically competitive system. This aligns with recent research suggesting that self-directed learning readiness can positively mediate learning outcomes regardless of students’ cultural orientation (Wang et al., 2021).

Further, the Chinese shift toward promoting more student-centered learning, adaptation of blended and online formats, and increasing emphasis on learners’ autonomy (as seen in policy directions and digital learning environments) means that measurement tools like the SDLS are timely and valuable.

## 5 Limitations

While this study offers valuable initial psychometric evidence for the SDLS among Chinese middle school students, several important limitations must be acknowledged. First, the participants were drawn exclusively from middle schools in Hebei Province and were all of Han ethnicity, which limits the extent to which findings can be generalized to other regions, ethnic groups, school types, or international contexts. Expanding the sample to include more diverse educational and demographic backgrounds was not realistic at this stage, but it is strongly recommended for future investigations. Second, measurement invariance across gender and grade was not examined due to limited subgroup sample sizes. Future research should test configural, metric, and scalar invariance using multi-group CFA with ΔCFI and ΔRMSEA criteria (Cheung and Rensvold, 2002; Chen, 2007). Third, this study’s cross-sectional design precluded assessment of test–retest reliability and the inclusion of external criterion measures (e.g., school performance, teacher ratings, observed learning behavior). Without these, claims about temporal stability and predictive validity of the SDLS are constrained; future research should incorporate longitudinal follow-ups and relevant criterion data. Fourth, the SDLS primarily measures students’ self-reported perceptions of motivation and autonomy, rather than directly observed learning behaviors or outcomes; responses may be influenced by social desirability or recall bias. Given that the data were collected in a performance-oriented educational context that strongly values academic achievement and teacher approval, students may have tended to provide socially desirable responses that align with expected “motivated” or “self-disciplined” behaviors. Additionally, Items 5 and 9 may capture somewhat distinct dimensions—values orientation and social comparison—which, although theoretically relevant to self-directed learning, could introduce minor heterogeneity within the construct. This potential content variation did not appear to undermine the unidimensional model in the present analysis but warrants further investigation through item-level analyses or bifactor modeling to confirm construct validity. Future studies could include validity checks such as impression management scales or triangulation with teacher or peer ratings to mitigate these possible biases and further validate the construct. Finally, correlations with theoretically related constructs (such as academic self-efficacy, self-regulated learning, or past academic achievement) were not assessed, limiting analysis of convergent and discriminant validity. Future studies should include such constructs to establish stronger validity connections.

## 6 Practical implications

Given the psychometric soundness of the SDLS, educators and school administrators can consider the following applications: (1) The SDLS can help identify students with lower self-initiative or autonomy; teachers can then offer targeted scaffolding (e.g., guided planning, resource-finding tips, self-monitoring strategies) to help those students gradually develop more self-directed habits. (2) When designing modules or tasks requiring independent or asynchronous work, the SDLS could be administered beforehand to gauge how well students are likely to perform. This may guide the structuring of teacher support, peer collaboration, feedback timing, or scaffolding of autonomy. (3) At the broader level, aggregated SDLS data from multiple classes or schools could reveal trends in self-directed learning readiness. This feedback may support pilot programs aimed at strengthening student agency, planning, goal-setting, and resource-use skills.

## 7 Conclusion

The Chinese version of the SDLS demonstrated sound psychometric properties among middle school students, with strong reliability, satisfactory content and structural validity, and a verified single-factor model. It provides a culturally appropriate and methodologically sound instrument for assessing self-directed learning and offers valuable implications for educational psychology research and practice in the evolving Chinese school context.

## Data Availability

The raw data supporting the conclusions of this article will be made available by the authors, without undue reservation.
