# Supplementary material for: Reliability and validity testing of the Chinese version of the self-directed learning scale among middle school students
Source: Front Psychol. 2025 Nov 25;16:1642596. doi: 10.3389/fpsyg.2025.1642596 (PMC12685800; doi:10.3389/fpsyg.2025.1642596)
Supplement: Supplementary file 1 [file Table_1.docx]

Supplementary Material

# Supplementary Data

**自主学习量表中文版**

**(Chinese Version of the Self-Directed Learning Scale)**

受访者须采用五点李克特量表来表明同意程度 (Respondents are required to indicate their level of agreement using a five-point Likert scale).

1 = 非常不同意 (Strongly Disagree).

2 = 比较不同意 (Somewhat Disagree).

3 = 中立 (Neutral).

4 = 比较同意 (Somewhat Agree).

5 = 非常同意 (Strongly Agree).

1. 我经常在课外自学 (I regularly learn things on my own outside of class)._____

2. 对于老师在课堂上没有解释的内容，我非常擅长自己寻找答案 (I am very good at finding out answers on my own for things that the teacher does not explain in class). _____

3. 如果课堂上有我不理解的内容，我总会找到自学的方法 (If there is something I do not understand in class, I always find a way to learn it on my own). _____

4. 我善于寻找合适的资源来帮助我在学校取得好成绩 (I am good at finding the right resources to help me do well in school). _____

5. 我认为基于主动性的自主学习对于在学校和未来职业生涯中的成功非常重要 (I view self-directed learning based on my own initiative as very important for success in school and in my future career). _____

6. 我为要学习的内容设定自己的目标 (I set my own goals for what I will learn). _____

7. 我喜欢掌控自己学习的内容和时间 (I like to be in charge of what I learn and when I learn it). _____

8. 如果我需要学习某些东西，我会立即找到方法 (If there is something I need to learn, I find a way to do so right away). _____

9. 我比大多数学生更擅长自学 (I am better at learning things on my own than most students). _____

10. 我非常有动力自学，不必依赖其他人 (I am very motivated to learn on my own without having to rely on other people). _____

**
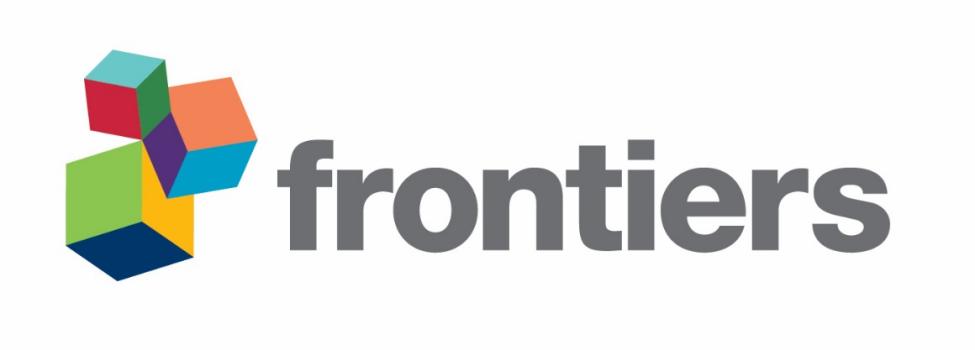
**
